# Patient Education for Endoscopic Sinus Surgery: Preliminary Experience Using 3D-Printed Clinical Imaging Data

**DOI:** 10.3390/jfb8020013

**Published:** 2017-04-07

**Authors:** Ian M. Sander, Taimi T. Liepert, Evan L. Doney, W. Matthew Leevy, Douglas R. Liepert

**Affiliations:** 1 Department of Biological Sciences, University of Notre Dame, South Bend, IN 46556, USA; isander@alumni.nd.edu (I.M.S.); edoney@alumni.nd.edu (E.L.D.); matthew.leevy@gmail.com (W.M.L.); 2 Michiana Sleep Solutions and Allied ENT Speciality Center, South Bend, IN 46635, USA; ttliepert@gmail.com

**Keywords:** 3D printing, sinonasal anatomy, clinical education, ENT

## Abstract

Within the Ear, Nose, and Throat (ENT) medical space, a relatively small fraction of patients follow through with elective surgeries to fix ailments such as a deviated septum or occluded sinus passage. Patient understanding of their diagnosis and treatment plan is integral to compliance, which ultimately yields improved medical outcomes and better quality of life. Here we report the usage of advanced, polyjet 3D printing methods to develop a multimaterial replica of human nasal sinus anatomy, derived from clinical X-ray computed tomography (CT) data, to be used as an educational aid during physician consultation. The final patient education model was developed over several iterations to optimize material properties, anatomical accuracy and overall display. A two-arm, single-center, randomized, prospective study was then performed in which 50 ENT surgical candidates (and an associated control group, *n* = 50) were given an explanation of their anatomy, disease state, and treatment options using the education model as an aid. Statistically significant improvements in patient ratings of their physician’s explanation of their treatment options (*p* = 0.020), self-rated anatomical understanding (*p* = 0.043), self-rated understanding of disease state (*p* = 0.016), and effectiveness of the visualization (*p* = 0.007) were noted from the population that viewed the 3D education model, indicating it is an effective tool which ENT surgeons may use to educate and interact with patients.

## 1. Introduction

Communication with patients has long been regarded as one of the most critical yet challenging parts of a doctor’s clinical work [1]. Patient understanding of their diagnosis and treatment regimen is integral to individual compliance and satisfaction with their physician [1,2,3]. Visual aids can be an important clinical tool for educating patients as they tend to understand and retain information more effectively with visual reinforcement [3]. However, classical graphical aids, such as photographs or illustrations, often require patients to view a three-dimensional morphology in a two-dimensional representation. Patient comprehension can be especially difficult with regards to internal anatomic relationships such as the nasal sinus passages, as these are not commonly visualized systems. Poor comprehension can hinder the informed consent process and may result in the patient electing not to move forward with a beneficial and necessary procedure. This is particularly prevalent in the Ear, Nose, and Throat (ENT) medical space, where surgery cancelation rates exceed 16% [4]. Therefore, advanced teaching aids may improve patient understanding and consent, leading to improved clinical outcomes.

Three-dimensional (3D) printing has emerged as an invaluable tool for the creation of anatomical models. The intricate complexity that can be replicated with advanced additive technology has enabled the synthesis of patient-specific replica models and vastly increased the wealth of educational and pre-surgical training models currently available [5]. Further driving the innovation in this space are multi-head 3D printers that enable several polymers with varying properties, such as color and hardness, to be mixed in a 3D space within the same model [6]. In particular, the Objet PolyJet™ systems use acrylic polymer substrates with variable side-chain groups to achieve tunable material properties [7]. These materials can be mixed at customizable ratios from each extruder head, at specific spatial coordinates, to yield novel composite models. While the use of 3D printing has been reported in the areas of oncology [8], craniofacial reconstruction [9], orthopedics [10], and organ models [11,12,13], there has been relatively little work reported in the rhinology/otolaryngology space [14]. Here we report the use of PolyJet technology to create an anatomical replica of patient sinus passages, with soft tissue and bone structure represented with malleable and hard polymeric materials, respectively. These customized models can be opened to allow patients to explore and manipulate the nasal sinus anatomy so that they may have a comparable teaching aid to those used in medical school anatomy labs. ENT doctors employed the models as teaching aids to evaluate their ability to improve patients’ self-reported understanding of their anatomy, disease state, and proposed surgical treatment versus traditional clinical education tools.

## 2. Results

### 2.1. Model Fabrication

**Figure 1:**
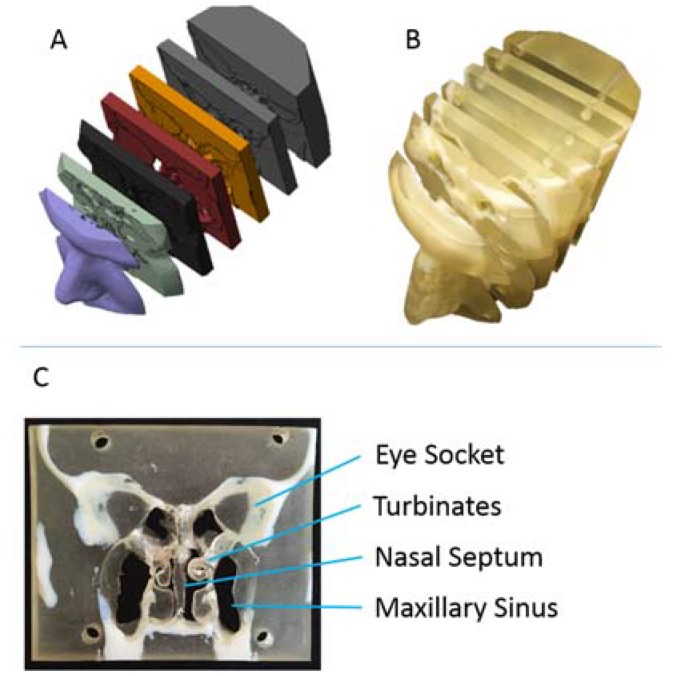
Nasal cavity model generation and fabrication. (**A**) 3D surface maps generated from CT scan slices of patient nasal cavity used to generate a 3D-printed model; (**B**) 3D-printed, sliced model, printed with two distinct polymers to represent hard and soft tissues; (**C**) Single coronal model slice with specific anatomical details highlighted.

A 3D-printed model was generated from X-ray computerized tomography (CT) scans of the nasal sinus anatomy of a patient (Figure 1A). The model was printed using a malleable, translucent polymer to represent soft tissue areas and a stiff, opaque polymer for bone tissue regions. Itwas cut into seven coronally-sliced slabs, which could be disassembled to expose and show the interior sinus and nasal anatomy (Figure 1B). The turbinates and nasal septum, soft nasal tissues, were accessible and easily manipulated by the ENTs to demonstrate physical manifestations of the patient’s disease state, altered air flow pathways, and proposed surgical treatments to the patients (Figure 1C). In this fashion, a single model was adaptable to describe the disease states of all individuals, preventing the need to create expensive, patient-specific repicas on a case-by-case basis.

### 2.2. 3D-Printed Model as an Educational Tool

One hundred adult candidates for sinus or nasal surgery were enrolled in a randomized clinical study to measure the efficacy of anatomically accurate, 3D-printed models in improving patient understanding of their anatomy, disease state, and surgical options. Each patient received an explanation of their condition from their ENT physician, either with a 3D-printed teaching model generated from CT scan data or with standard diagrams and charts. Immediately following the ENT consultation, each patient was given a survey to rate the physician’s explanation of the proposed treatment plan, their own understanding of their anatomy and disease state, and how much the educational tools aided the physician’s explanation. Patients were also asked if the ENT consultation eased their anxiety and if they intended to go through with the proposed treatment plan, if applicable. Patient ratings for questions 1–5 were averaged and subjected to a non-parametric Mann-Whitney test comparing the 3D model and control groups. Patient responses for questions 6 and 7 were collected and the percentage of “yes” responses was calculated and subjected to a chi-squared test to determine the statistical significance. “N/A” responses in question 6 were removed when calculating the percentage of “yes” responses. The survey questions and averaged results are shown in Table 1.

The average score for each survey question was higher for the 3D model group compared to the control group. Patient responses in the 3D model group increased significantly when rating the quality of the physician’s explanation, their own understanding of their anatomy, their own understanding of their disease state, and their understanding of the proposed surgical treatment. Averages in both groups approached the ceiling score, which may impact the significance of the results. The largest increase in score was seen in patient understanding of the surgical treatment, which is the most critical information to ensure the patient is making an informed decision. Interestingly, 32 patients said the standard visual aids eased their anxiety, while 39 responded positively to the 3D model. Questions 6 and 7, which were more qualitative in nature, did not result in statistically significant results between the control and 3D model groups, though a higher percentage of patients did respond in the affirmative to each question when presented with the 3D model. Overall, the survey results demonstrate the positive educational benefits of the 3D model as a clinical teaching tool for ENTs.

## 3. Discussion

The survey data imply that providing patients with a 3D model during their physician consultation is equivalent or superior to the current clinical standard for improving patient self-reported understanding of their anatomy, condition, and treatment options. The results of the survey are notable for a variety of reasons. First, although the scores in the 3D model experimental group were higher across the board, the control group still showed high self-reported scores for each question. On one hand, this suggests that the standard explanation and tools ENTs use provide their patients with a very good overall understanding of their health, anatomy, condition, and treatment options. On the other hand, since these scores are all self-reported, it may convolute the true benefits of improved educational models since so many patients already reported perfect or near-perfect understanding. Additionally, the abundance of high scores clustered close to the maximum value (10) could impact the ability to identify truly significant changes. Future studies with larger cohorts in each group could help in distinguishing which increases are significant, and could also test each patient to objectively measure the understanding of their anatomy. Overall, it was not surprising to see high self-reported survey results as the two ENT physicians, who each saw a randomly distributed subset of patients from each cohort, were highly experienced, with five and 20 years of experience, respectively. A greater impact from use of the model would be hypothesized for less experienced ENT doctors, or perhaps from those in which English is a second language.

As mentioned above, the largest difference in score between the control and 3D model groups was in patient understanding of the surgical procedure. The cohort given the 3D model reported a nearly perfect understanding of the surgery, with an average score of 9.82 out of 10. Only one patient reported an understanding level of 8, seven reported 9, and the remaining 42 patients reported a maximum understanding of the surgical procedure. By comparison, the control group had eight reports of scores 8 or below. Given that physicians use educational aids to inform their patients about the details of the proposed surgical treatment, the 3D models appear to be an improved tool for these means.

On the whole, the degree of patient teaching varies between doctors. However, a common thread between all doctors is their desire to improve patient outcomes. Physicians may be less likely to explain difficult and complex ideas if they are concerned the information may be misunderstood and may lead patients to opt out of beneficial treatment options due to fear or lack of comprehension. Patient anxiety has been reported as a significant hurdle to opting for a surgical procedure [15]. However, in this study, the use of the 3D models was found to ease patient anxiety, with 97.5% of respondents reporting reduced apprehension, as well as increase self-reported understanding. Understanding of the surgical procedure is critical to patient-centered care models and well-informed individuals are better equipped to make decisions about treatment options that are suitable for them.

The results from the patient survey show a trend pointing at the benefits of realistic, interactive teaching models for ENTs. Future work could include additional patient surveys conducted both prior to their physician consultation and at a set period of time after the consultation. This would provide additional insights into the efficacy of the 3D model teaching tools to improve comprehension, understanding, and retention of information regarding the patient condition and treatment options. To get a better measure of patient understanding relative to self-reporting, a quiz could be designed and administered to patients to gather quantitative results, as has been used in other studies to measure patient understanding [16,17].

## 4. Materials and Methods

### 4.1. Selection of Patients and Study Design

Prior to recruiting patients, this study was approved by the University of Notre Dame’s Institutional Review Board. IRB approval was granted for waiver of written informed consent for this study due to minimal risk and the need to prevent full disclosure that may have induced bias. Each patient received a letter describing the study and agreed to participate. The letter stated: “Dr. (Insert physician name) is participating in a new research study to determine which explanation techniques will help patients to better understand their nasal and sinus anatomy, the disease they have been diagnosed with, and the surgical choices they are being given. You have been asked to participate in this research study because your doctor has recommended nasal or sinus surgery. This study consists of your disease, anatomy and surgical procedure being described to you both verbally and using other forms of education materials. Patients in this study are randomly assigned to one of two groups. The description will be done using different explanation techniques for each group. This will be followed by a seven question survey that you will be asked to fill out. The sponsor of this study is the Notre Dame Biological Imaging Laboratory. The sponsor is paying your surgeon to participate in this study to help cover the activities involved in this study. Participation in this research study is voluntary. You can withdraw from this study at any time. Your participation does not in any way affect your treatment by your physician. Please let your physician know if you would like to participate. Thank you for considering participation in this new research study.” 

The study was a prospective, two arm, single center, randomized study. The randomization of patients was determined using a computational random number generator, with patients entering the study via their surgeon. From February 2015 to April 2015, 100 adult patient candidates for sinus or nasal surgery were included, each aged 18 years or older.

Patients were verbally informed about the study design and were given a letter with the highlights. They were randomized into either the study or control group, with 50 patients in each arm. The patient’s disease, anatomy, and surgical options were explained in a method consistent with their cohort assignment. The control group had the information about their anatomy, disease, and surgical option(s) explained by their surgeon using the current standard of clinical care, including the use of 2D charts and a verbal explanation. The study group had the same information given to them using an anatomically accurate, 3D printed model of the nasal/sinus region of a standard head to help explain their anatomy, disease, and surgical option(s). The same model was used throughout the study. After this explanation, a seven-question survey was given to each patient to complete. There was no time restriction given to the patient to fill out the survey, and the patient understood that their surgeon would not see the answers to their survey.

The survey results for each patient were collected and analyzed in conjunction with the other patients in their respective assigned cohort. For questions 1–5, a median patient score for each cohort was calculated and compared to the other cohort using an upaired, non-parametric Mann-Whitney test. Results were taken to be statistically significant for *p* < 0.05. For question 6, the number of patients answering “yes,” “no,” and “not applicable” were totaled. The percentage of patients answering “yes” in each cohort was calculated by summing the number of “yes” responses and dividing by the total number of “yes” and “no” responses; “not applicable” responses were excluded from the calculation. For question 7, the number of patients answering “yes,” “no,” and “not sure” were totaled. The percentage of patients answering “yes” in each cohort was calculated by summing the number of “yes” responses and dividing by the total number of responses. No patient answered “no” to question 7. For questions 6 and 7 the percentage of “yes” answers were compared between cohorts with a chi-squared test, with *p* < 0.05 set as the cutoff for determining statistical significance.

### 4.2. CT Data Processing and 3D Printing

Pre-existing cone beam CT data acquired with a MiniCAT (Xoran Technologies, Ann Arbor, MI, USA) was used for 3D printed model generation. The CT data was opened in Pmod 3.2 Biological Image Quantification software (Pmod Technologies, Zurich, Switzerland) with the View tool. A volume of interest was drawn around the portion of the scan that included the nose, nasal cavity, and sinuses, but excluded the teeth and other tissue at the edge of the scan. All volumes outside of the volume of interest were masked at a value of −1000 Hounsfield Units (HU). The data was exported in Nifti (.nii) format and opened in 3DSlicer [18].

A 3D surface map for bone was generated using the “Grayscale Modelmaker” tool within 3DSlicer at a threshold of 300 HU. A separate 3D surface map was generated for soft tissue using the same tool and process with a threshold level of −300 HU. Each surface map was exported from 3DSlicer as a .stl file. Soft tissue and bone 3D surface maps were imported separately into MeshLab software (ISTI–CNR) [19]. Within MeshLab, the “Remove Isolated Pieces” algorithm was applied to each surface map with a threshold of 10% to remove unconnected surfaces from the overall 3D surface map. The “Laplacian Smooth” algorithm was applied to each surface map in three steps. Smoothed surface maps were then exported as .stl files.

Smoothed bone and soft tissue surface maps were imported into Autodesk Netfabb software (Autodesk, Inc., San Rafael, CA, USA) into the same session [20]. Cuts were made on the coronal plane starting at the plane of the canthi and continuing posteriorly at intervals of 1 cm, until the sphenoid sinus was bisected by at least one cut. A total of 6 cuts were made in the model used for this study, resulting in 7 slices. Cuts were not triangulated, and both parts were aligned to cut at the same time. After the cuts were made, each slab of the model was taken into “Repair” mode in Netfabb, holes were bridged with the “Add Triangles” tool, and the “Automatic Repair” script was applied. Each slab was exported from Netfabb as a separate .stl file. 

The .stl files were imported into Objet Studio (Stratasys, Ltd., Eden Prairie, MN, USA), aligned, and assigned materials. TangoPlus (Shore A: 27) was used for the soft tissue, and VeroWhite was used for bone (Shore D: 83). Models were printed on an Objet Connex500 3D Printer with a glossy finish. Support material was removed via water jet without disrupting the anatomy prior to delivery to the ENT clinic.

## 5. Conclusions

Three-dimensional printing is a powerful tool that is just beginning to find its uses in a variety of areas. New examples of its applications in healthcare are occurring at a rapid pace. Here, we have demonstrated the use of a 3D-printed model of the nasal sinus anatomy as an educational aid for ENTs to teach patients about their anatomy, medical conditions, and surgical treatment options. Across the entire breadth of survey questions given to candidates for nasal surgery, patients reported better understanding and comfort levels when their ENT included a 3D-printed model as part of their consultation compared to being educated with the current standard of 2D diagrams and drawings. The patients’ ability to better visualize their condition and potential treatment options led to self-reported greater understanding, improved comprehension, and less anxiety, all of which can contribute to better patient outcomes through informed consent and increased willingness to go through with beneficial procedures.

## Figures and Tables

**Table 1:** Sinus surgery patient survey questions and average response scores after ENT consultation.

Survey Question	Control Score (±St Dev)	3D Model Score (±St Dev)	*p*-Value
How well did your doctor explain the surgery? (1–10):	9.38 (0.80)	9.70 (0.65)	0.020 *
Rate your understanding of the surgery after your doctor’s explanation (1–10):	9.14 (0.86)	9.40 (0.88)	0.078
Rate your understanding of your anatomy after your doctor’s explanation (1–10):	8.86 (1.29)	9.32 (0.91)	0.043 *
Rate your understanding of your disease after your doctor’s explanation (1–10):	9.06 (1.28)	9.48 (0.99)	0.016 *
How did the doctor’s visualization help you in your understanding of the surgery? (1–10):	9.32 (1.11)	9.82 (0.44)	0.007 *
Did the explanation ease your anxiety? (Y, N, N/A):	91.4% Y ^1^	97.5% Y ^2^	0.243
Do you plan on getting the recommended surgery? (Y, N, not sure):	88% Y ^3^	90% Y ^4^	0.749

^1^ 32 patients responded “yes,” three responded “no,” 15 responded “N/A”; ^2^ 39 patients responded “yes,” one responded “no,” 10 responded “N/A”; ^3^ 44 patients responded “yes,” zero responded “no,” six responded “not sure”; ^4^ 45 patients responded “yes,” zero responded “no,” five responded “not sure”. * *p* < 0.05 considered statistically significant.
